# In vivo 3D myocardial membrane potential mapping in humans using PET/MRI

**DOI:** 10.1186/s13550-025-01287-7

**Published:** 2025-07-26

**Authors:** Felicitas J. Bijari, Paul Kyu Han, Thibault Marin, Wonil Lee, Yanis Chemli, Inna Gertsenshteyn, Ismaël B. G. Mounime, Yanis Djebra, Didi Chi, Marc D. Normandin, Chao Ma, Georges El Fakhri

**Affiliations:** 1 Yale Biomedical Imaging Institute, Yale University School of Medicine, New Haven, CT USA; 2 Department of Radiology and Biomedical Imaging, Yale University School of Medicine, New Haven, CT USA; 3 Department of Biomedical Informatics and Data Sciences, Yale University, New Haven, CT USA; 4 Independent Researcher, Boston, MA USA; 5 LTCI, Télécom Paris, Institut Polytechnique de Paris, Palaiseau, France

**Keywords:** Membrane potential mapping, Positron emission tomography, Magnetic resonance imaging, 3D

## Abstract

**Background:**

The mitochondrial membrane potential is a key biophysical parameter of mitochondrial function*,* which can be useful for the diagnosis and treatment monitoring of various cardiac diseases. We present a non-invasive PET/MR imaging method for 3D myocardial membrane potential mapping in humans.

**Results:**

An in vivo PET/MR imaging study was performed in three healthy subjects (1 male and 2 females; 48 ± 29 years old) under a study protocol approved by the local Institutional Review Board (IRB). Written informed consent was obtained from all subjects before participation in the study. The [^18^F](4-Fluorophenyl)triphenylphosphonium ([^18^F]-FTPP^+^) PET tracer was administered using a bolus-plus-infusion protocol (bolus activity of 301.2 ± 7.6 MBq, infusion activity of 90.0 ± 4.9 MBq), where an infusion of 120 min was started shortly after the bolus injection (time of infusion, TOI). Dynamic cardiac PET/MR imaging was performed approximately 20 min after the TOI and continued for 100 min. The extracellular volume fraction mapping was performed via cardiac MR with a free-breathing, 3D cardiac *T*_*1*_ mapping sequence before and after the contrast agent injection (gadoterate meglumine, 0.1 mmol/kg). A linear tangent space alignment (LTSA) model-based method was used to reconstruct high-frame-rate dynamic images from sparsely sampled (*k,t*)-space data for *T*_*1*_. PET motion correction was performed using two steps of rigid image registration in a multi-resolution fashion, followed by a non-rigid image registration with B-spline transform. The tissue membrane potential was calculated using a kinetic model based on the Nernst equation with myocardial tracer concentration, tracer volume of distribution, and extracellular volume fraction measurements. Fully 3D membrane potential maps were successfully estimated from all three subjects. The estimated whole-heart membrane potentials were − 144.7 ± 3.5 mV, − 160.7 ± 5.3 mV, and − 165.8 ± 3.1 mV for each subject.

**Conclusion:**

The proposed method allows 3D myocardial membrane potential mapping in humans in vivo.

**Supplementary Information:**

The online version contains supplementary material available at 10.1186/s13550-025-01287-7.

## Introduction

Mitochondrial dysfunction plays a key role in many cardiac diseases, such as heart failure, ventricular arrhythmias, or chemotherapy-induced cardiotoxicity [1–3]. The mitochondrial membrane potential established by the proton gradient across the inner mitochondrial membrane provides the energy for mitochondrial adenosine triphosphate (ATP) generation and is a comprehensive index of mitochondrial function. Consequently, non-invasive quantification of myocardial mitochondrial membrane potential allows the assessment of mitochondrial function in vivo*,* which can be useful for the diagnosis and treatment monitoring of cardiac diseases.

Measuring mitochondrial membrane potential in vitro has been possible for decades with lipophilic cationic probes, such as 3H-tetraphenylphosphonium (TPP^+^) or fluorescent dyes like tetramethylrhodamine ethyl ester [4]. However, in vivo measurement of mitochondrial membrane potential is not yet available as a standard method and is an active area of research. Several attempts have been made to measure mitochondrial membrane potential in vivo with positron emission tomography (PET) [5–7]. However, these methods assumed radiotracer activity to be at secular equilibrium with a bolus injection of the radiotracer, which is not a valid assumption. Recently, our group has developed an in vivo imaging method that measures the total membrane potential, a proxy of mitochondrial membrane potential, in absolute units of millivolts (mV) using [^18^F]4-fluorophenyltriphenylphosphonium ([^18^F]-FTPP^+^) PET with a bolus plus infusion injection of the radiotracer [8–10]. As previously described in [8, 9], the method requires the measurement of both the volume of distribution of [^18^F]-FTPP^+^ and the extracellular volume fraction, which can be measured using CT or MR imaging with contrast agent injection [9].

Of note, in vivo myocardial mitochondrial membrane potential mapping has been demonstrated for the first time in healthy subjects with simultaneous [^18^F]-FTPP^+^ PET and MR imaging [10]. However, this work used a breath-holding, electrocardiogram (ECG)-triggered 2D cardiac T1 mapping sequence [11] to obtain the extracellular volume fraction, which necessitates multiple breath-hold scans for extracellular volume fraction mapping of the whole heart. The limitations of this breath-hold cardiac *T*_*1*_ mapping approach include inapplicability in dyspneic patients (e.g., patients with chronic cardiotoxicity), poor image quality with problematic breath-holding, and inadequate through-plane spatial resolution and volume coverage. Multiple breath-holds can be problematic for patients in general, especially those with severe cardiac disease.

In this work, we present a non-invasive PET/MR imaging method that enables full 3D myocardial membrane potential mapping in humans. We extended the approach developed by Pelletier-Galarneau et al. by utilizing a recently developed MR-based, free-breathing, 3D extracellular volume fraction mapping method [12]. Simultaneous, dynamic cardiac PET/MR imaging was performed with a bolus-plus-infusion injection of [^18^F]-FTPP^+^. Three-dimensional (3D) extracellular volume fraction mapping was performed using a free-breathing, ECG-triggered 3D cardiac *T*_*1*_ mapping sequence [13] before and after the administration of a gadolinium-based contrast agent. MR image reconstruction was performed using a linear tangent space alignment (LTSA) model-based framework [14], to allow the recovery of high frame-rate dynamic MR images in real-time from highly under-sampled data. The 3D myocardial membrane potential map was generated using a kinetic model based on the Nernst equation with voxel-wise extracellular volume fraction measurements, myocardial tracer concentration at secular equilibrium, and plasma tracer concentration. We demonstrate the feasibility of the proposed method by presenting our initial results on 3D myocardial membrane potential mapping in healthy volunteers.

## Material and methods

### Data acquisition

**Fig. 1:**
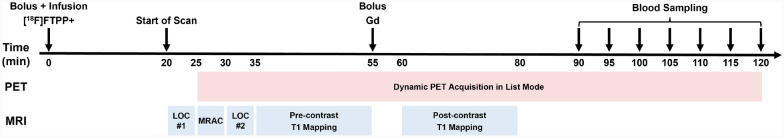
Schematic diagram of the proposed 3D membrane potential mapping method

An in vivo PET/MR imaging study was performed with three healthy subjects (1 male and 2 females; 48 ± 29 years old) under a study protocol approved by the Mass General Brigham Institutional Review Board (IRB). Written informed consent was obtained from all subjects before participation in the study. [^18^F]-FTPP^+^ was administered using a bolus-plus-infusion protocol (bolus activity of 301.2 ± 7.6 MBq, infusion activity of 90.0 ± 4.9 MBq) where an infusion of two hours was started shortly after the bolus injection (time of infusion, TOI). Dynamic cardiac PET/MR imaging was performed on a 3 T PET/MRI scanner (Biograph mMR, Siemens, Erlangen, Germany) as shown in Fig. 1. PET imaging was performed around 25 min after the TOI and continued for 95 min. MR imaging was performed around 20 min after the TOI for 3D extracellular volume fraction mapping similar to a previous study [12]: a 3D free-breathing cardiac *T*_*1*_ mapping sequence [13] was used before and after the contrast agent injection (gadoterate meglumine, 0.1 mmol/kg). The imaging parameters were: matrix size = 160 $\times$ 160 $\times$ 32, spatial resolution = 1.9 $\times$ 1.9 $\times$ 4.5 mm^3^, flip angle = 9°, TR/TE = 4.2/1.7 ms, ECG-triggered acquisitions with 10-(3)-10-(3) protocol [13], and frame-rate per 3D volume = 138.6 ms. A special data sampling scheme that under-samples the (*k,t*)-space data following random ordering of radial, stack-of-stars sampling trajectory was used [13]. A respiratory navigator signal was acquired at the center of the lung/liver interface for each frame to help track respiratory motion. The total scan time was 10.7 ± 1.5 and 10.1 ± 2.2 min for pre- and post-contrast acquisitions, respectively, with a pause time of 15.4 ± 4 min after the contrast agent injection. Additional 2D T_1_ maps were acquired using the standard 2D MOLLI method [11] before and after the contrast agent injection over the apical, mid-cavity, and basal regions of the heart for comparison. Serial venous blood samples were obtained every 5 min between 90 to 120 min after the TOI, and the tracer activity concentration in plasma was measured with a calibrated well counter.

### Image reconstruction

The dynamic MR images were reconstructed using the LTSA model [14], which leverages the underlying low-dimensional manifold structure of the dynamic MR signals for image reconstruction with highly under-sampled (*k,t*)-space data. More specifically, the image reconstruction was performed by solving the following optimization problem:

1 $$\arg \mathop {\min }\limits_{T,L}\parallel d - AX\parallel_{2}^{2} + \lambda_{{\text{L}}} \parallel vec\left( L \right)\parallel_{1} + \lambda_{{\text{T}}} \parallel {\mathcal{D}}\left( T \right)\parallel_{1} + \frac{{\mu_{{\text{T}}} }}{2}\parallel T\parallel_{F}^{2} + \frac{{\mu_{{\text{L}}} }}{2}\parallel L\parallel_{F}^{2} {\text{ s}}.{\text{t}}.{ }X_{c} = \mathop \sum \limits_{c = 1}^{C} TL_{c} {\Phi }_{c}^{T}$$

where $X\in {\mathbb{C}}^{J\times K}$ denotes the dynamic MR images with $J$ representing the number of image voxels and $K$ representing the number of frames (with frame-rate as described in Sect. “Data acquisition”), represented by the LTSA model [14]. In this model, ${X}_{c}$ represents the dynamic image set from a neighborhood $c$, which is defined as cardiac phases separate for pre- and post-contrast acquisitions, $T\in {\mathbb{C}}^{J\times R}$ represents the global coordinates with rank $R$, $L=[{L}_{1}, \dots ,{L}_{C}]\in {\mathbb{C}}^{R\times CR}$ represents the concatenation of the alignment matrices where ${L}_{c}\in {\mathbb{C}}^{R\times R}$ is the alignment matrix for the neighborhood $c$, ${\Phi }_{c}\in {\mathbb{C}}^{{K}_{c}\times R}$ represents the temporal bases for neighborhood $c$. The first term of the cost function penalizes the data fidelity with $d$ denoting the acquired (*k,t*)-space data and $A=\Omega {F}_{s}$ denoting the forward operator from image space to *k*-space, where $\Omega \left(\bullet \right)$ is the sparse sampling operator, ${F}_{s}$ is the spatial Fourier transform operator implemented with the nonuniform fast Fourier transform (NUFFT) [15]. Additional sparsity constraints and ${{\ell}}_{2}$ regularization terms were introduced to promote piece-wise smoothness of the reconstructed images and to improve the robustness of the solution to noise perturbations [16], where $\mathcal{D}$ denotes the finite difference operator, ${\lambda }_{L}$, ${\lambda }_{T}$, ${\mu }_{L}$, and ${\mu }_{T}$ denote the corresponding weights.

The optimization problem in Eq. (1) was solved using the alternating direction method of multipliers (ADMM) method [17] with graphics processing units (GPUs) utilizing Python along with the CuPy library. The solver utilized the SigPy framework and the NUFFT on GPUs was conducted using the MRRT package. The reconstructions were performed on computing clusters equipped with four Tesla V100-SMX2 GPUs (16 GB memory each). The reconstructions were performed in parallel across GPUs for each coil. The total MRI image reconstruction time was 24.3 $\pm$ 5.2 min.

The dynamic PET images were reconstructed to 2.08 × 2.08 × 2.03 pixels and 1 min frame length using the ordered subset expectation maximization (OSEM) algorithm with point spread function (PSF) modeling (3 iterations, 21 subsets). Attenuation correction was performed using a vendor-provided MR-based attenuation correction method based on the Dixon sequence with model-based bone estimation. Scatter correction was performed using single scatter simulation [18].

### PET Motion correction and image registration

PET motion correction was performed on the reconstructed dynamic PET images in three successive steps using the SimpleITK library [19]. First, a mid-time frame from the 4D PET dataset was selected as the reference, and a spherical mask was defined around the heart to restrict the registration metric to that region. Two rigid registration passes were then carried out in a multi-resolution fashion (shrink factors of [4, 2, 1], smoothing sigmas of [2, 1, 0]), employing Mattes mutual information as the similarity metric and a conjugate gradient line search optimizer. The first pass used a relatively larger learning rate (0.001) and fewer iterations (1000) to coarsely align each frame to the reference, while the second pass utilized a smaller learning rate (0.0001) and more iterations (10,000) for finer adjustment. Finally, a third pass of non-rigid registration was performed using a B-spline transform (mesh size of [8, 8, 8]). The reconstructed PET and MR images were aligned to the same position in the short-axis view orientation based on the position information stored from the simultaneous PET/MRI acquisition. An additional rigid registration was performed to correct the remaining misalignment between the reconstructed PET and MR images.

### Estimation of myocardial membrane potential and analysis

Our membrane potential mapping approach relies on a compartmental model, including the extracellular space, cytosol, and mitochondria, for quantification of membrane potential in units of mV [8]. The volume of distribution of [^18^F]-FTPP^+^, and membrane potential, can be approximated by [8–10]:

2 $${V}_{T}=\frac{ \overline{PET} }{{\overline{C} }_{p} }\approx \left(1-{f}_{ECV}\right){\bullet f}_{mito}\bullet {e}^{-\beta \times \Delta {\Psi }_{T}}$$

3 $$\Delta {\Psi }_{T}=-\frac{1}{\beta }ln\left(\frac{{V}_{T}}{\left(1-{f}_{ECV}\right){\bullet f}_{mito}}\right)$$

where *V*_*T*_ denotes the tracer volume of distribution, $\overline{PET }$ (Bq/mL) denotes the activity concentration measured by the scanner at secular equilibrium, ${\overline{C} }_{p}$ denotes the equilibrium concentration in plasma (Bq/mL), ${f}_{ECV}$ (unitless) represents the extracellular volume fraction, ${f}_{mito}$ (unitless) represents mitochondrial volume fraction, $\beta =\frac{zF}{RT}$ is a known physical constant with *z* denoting the valence, *F* denoting the Faraday’s constant, *R* denoting the universal gas constant, and *T* denoting the temperature in degrees Kelvin, and $\Delta {\Psi }_{T}$ (mV) is the total membrane potential representing the sum of cellular and mitochondrial membrane potentials (i.e., $\Delta {\Psi }_{T}\triangleq \Delta {\Psi }_{c}+\Delta {\Psi }_{m}$). Because the mitochondrial membrane potential is much more negative than the cellular membrane potential, the total membrane potential can effectively serve as a proxy for the mitochondrial membrane potential.

The extracellular volume fraction was estimated for each voxel from the reconstructed MR images by first estimating the pre- and post-contrast ${T}_{1}$. The reconstructed MR images were binned into different respiratory motion phases based on the respiratory navigator signal. The *T*_*1*_ was then estimated with transmit *B*_*1*_ correction [13] for each voxel and respiratory motion phase, separately for pre- and post-contrast acquisitions, using a variable projection algorithm [20]. The *T*_*1*_ was estimated by finding the best fit between the signal from the reconstructed MR images and those from a dictionary of Bloch equation simulations. The dictionary of Bloch equation simulated signals was generated for a pre-contrast *T*_*1*_ range of 500–2500 ms with an increment of 10 ms, post-contrast *T*_*1*_ range of 200–1500 ms with an increment of 10 ms, and transmit B_1_ range of 0.2–1.5 with an increment of 0.05. For post-contrast acquisitions, additional signals were generated considering the *T*_*1*_ rate of change after contrast agent injection, for a range of 0–1 ms/s with an increment of 5 $\times$ 10^–2^ ms/s. The extracellular volume fraction was then calculated for each voxel using the following formula [21–23]:

4 $${f}_{ECV} \left(\text{\%}\right)=\frac{\Delta {R}_{1,r}}{\Delta {R}_{1,blood}}\times \left(1-HCT\right)\times 100 (\text{\%}),$$

where $\Delta {R}_{1,r}=1/{T}_{1,r,post}-1/{T}_{1,r,pre}$ denotes the difference in the reciprocal of pre- and post-contrast *T*_*1*_ at a voxel position $r$, $\Delta {R}_{1,blood}=1/{T}_{1,blood,post}-1/{T}_{1,blood,pre}$ denotes the difference in the reciprocal of pre- and post-contrast *T*_*1*_ from the blood pool region-of-interest (ROI), and *HCT* denotes the hematocrit level measured from the blood sample for each subject.

The voxel-wise tracer volume of distribution was determined from the reconstructed PET images by averaging myocardial and blood tracer activity concentration between 100 and 120 min post-TOI, when secular equilibrium was approximately reached in the myocardium and the venous plasma concentration is expected to represent the arterial plasma input function. The total membrane potential was calculated for each voxel using Eq. 3 with the estimated extracellular volume fraction, tracer volume of distribution, and *f*_*mito*_ of 0.25 [24]. The left ventricle of the heart from the extracellular volume fraction, tracer volume of distribution, and membrane potential maps were divided into 17 regions-of-interest (ROIs) according to the American Heart Association (AHA) 17-segment model [25] for analysis.

## Results

**Fig. 2:**
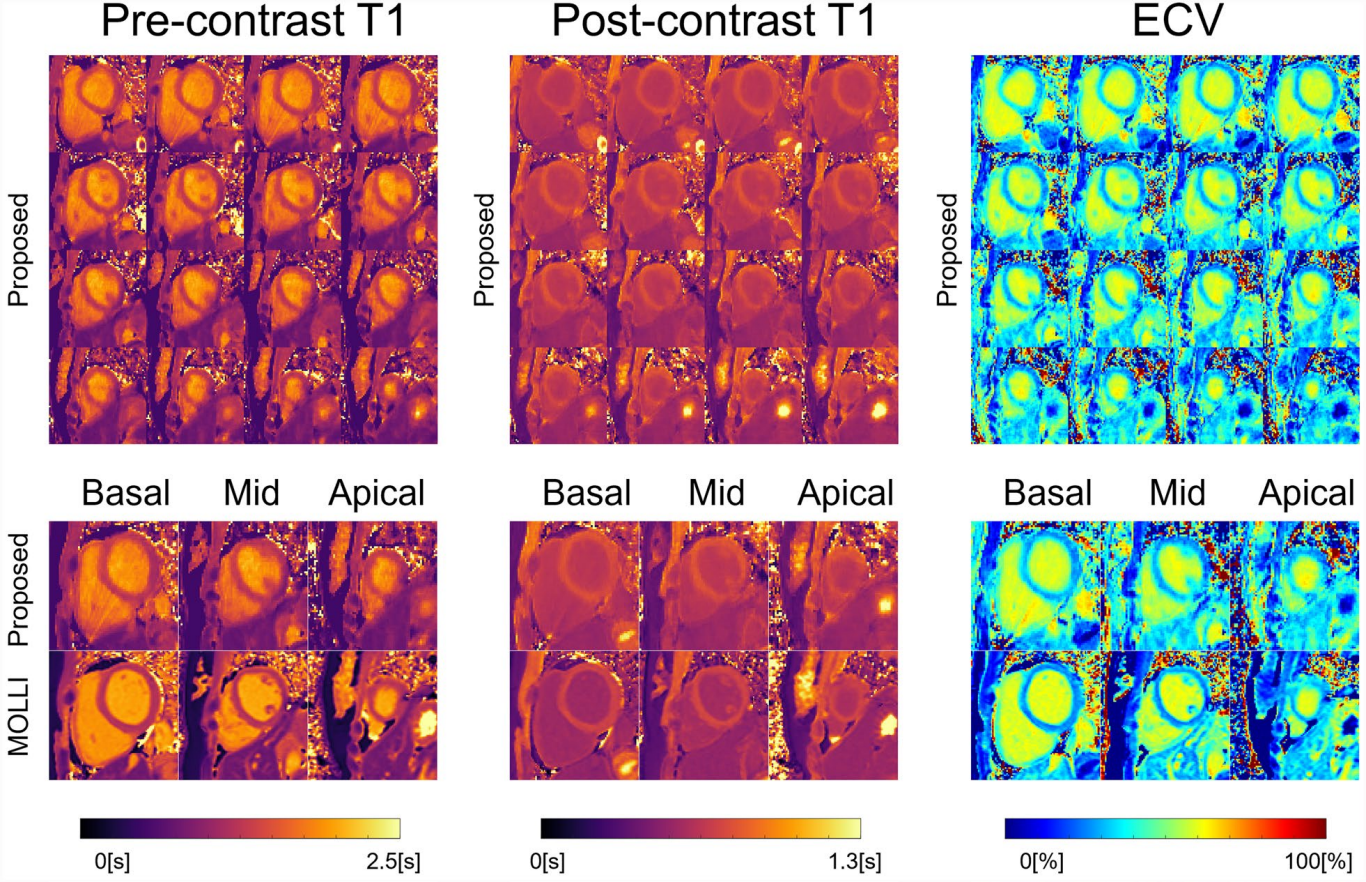
Pre-contrast *T*_*1*_, post-contrast *T*_*1*_, and extracellular volume fraction (*f*_*ECV*_) mapping results from a representative subject. Results of pre-contrast *T*_*1*_, post-contrast *T*_*1*_, and *f*_*ECV*_ maps are shown in 3D and in comparison with those from the reference MOLLI method at comparable slice regions

Figure 2 shows the pre-contrast *T*_*1*_, post-contrast *T*_*1*_, and extracellular volume fraction mapping results from the proposed method from a representative subject. Using the proposed method, the pre-contrast *T*_*1*_, post-contrast *T*_*1*_, and extracellular volume fraction maps were successfully acquired in 3D covering the whole heart. The pre-contrast *T*_*1*_, post-contrast *T*_*1*_, and extracellular volume fraction maps from the proposed method were qualitatively in good agreement with those from the reference 2D MOLLI method at the comparable slice regions.

**Fig. 3:**
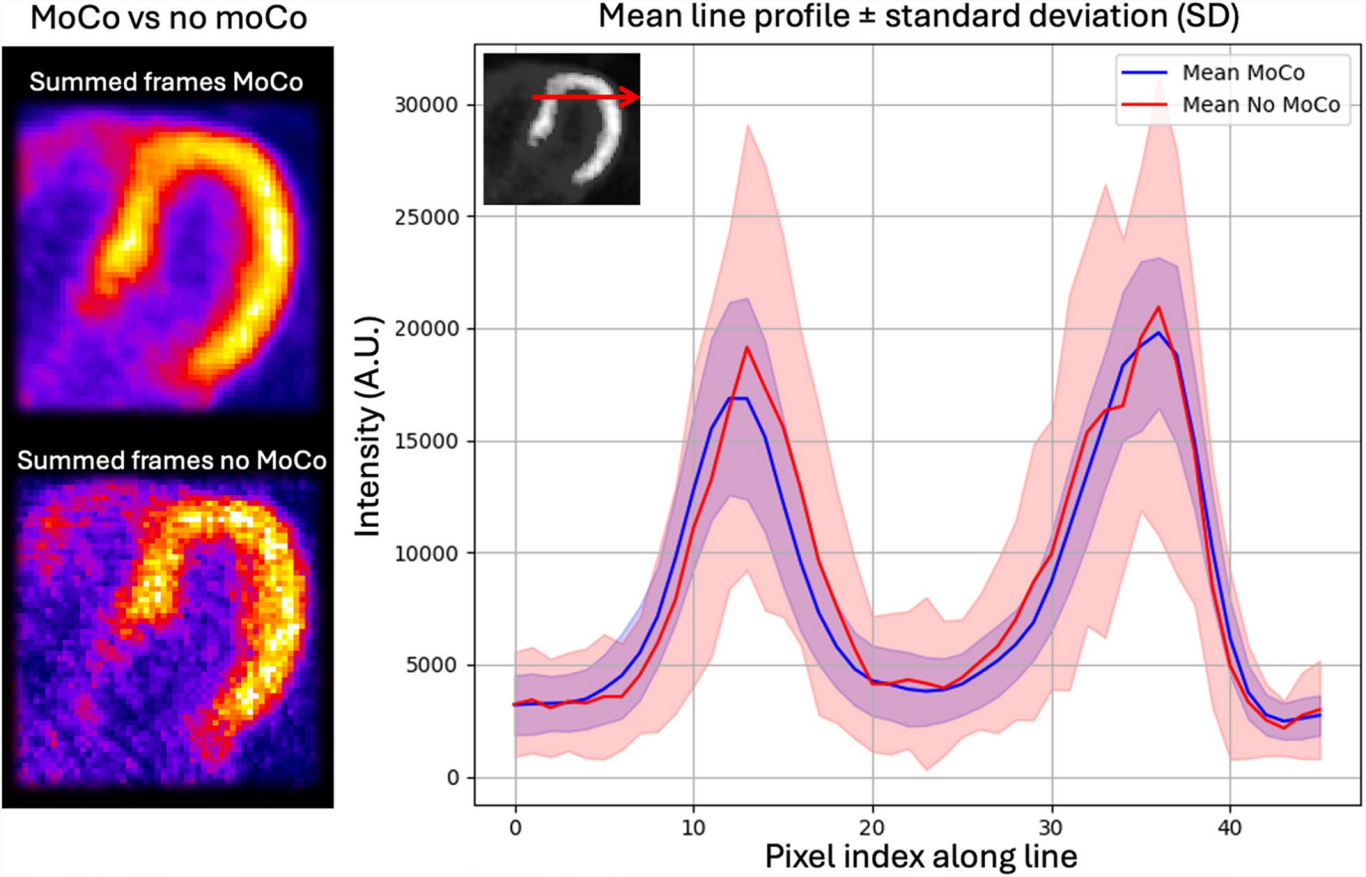
PET motion correction results from a representative subject. The summed dynamic PET images and a plot of the line profile across the left ventricle are shown for cases before and after motion correction. The shaded regions indicate the standard deviation over time frames, which is substantially reduced when motion correction is applied

Figure 3 shows the results of PET motion correction from the same representative subject. The summed dynamic PET images showed better delineation of the myocardium wall after motion correction. The line profile plot across the left ventricle showed reduced variability across the different dynamic frames after motion correction.

**Fig. 4:**
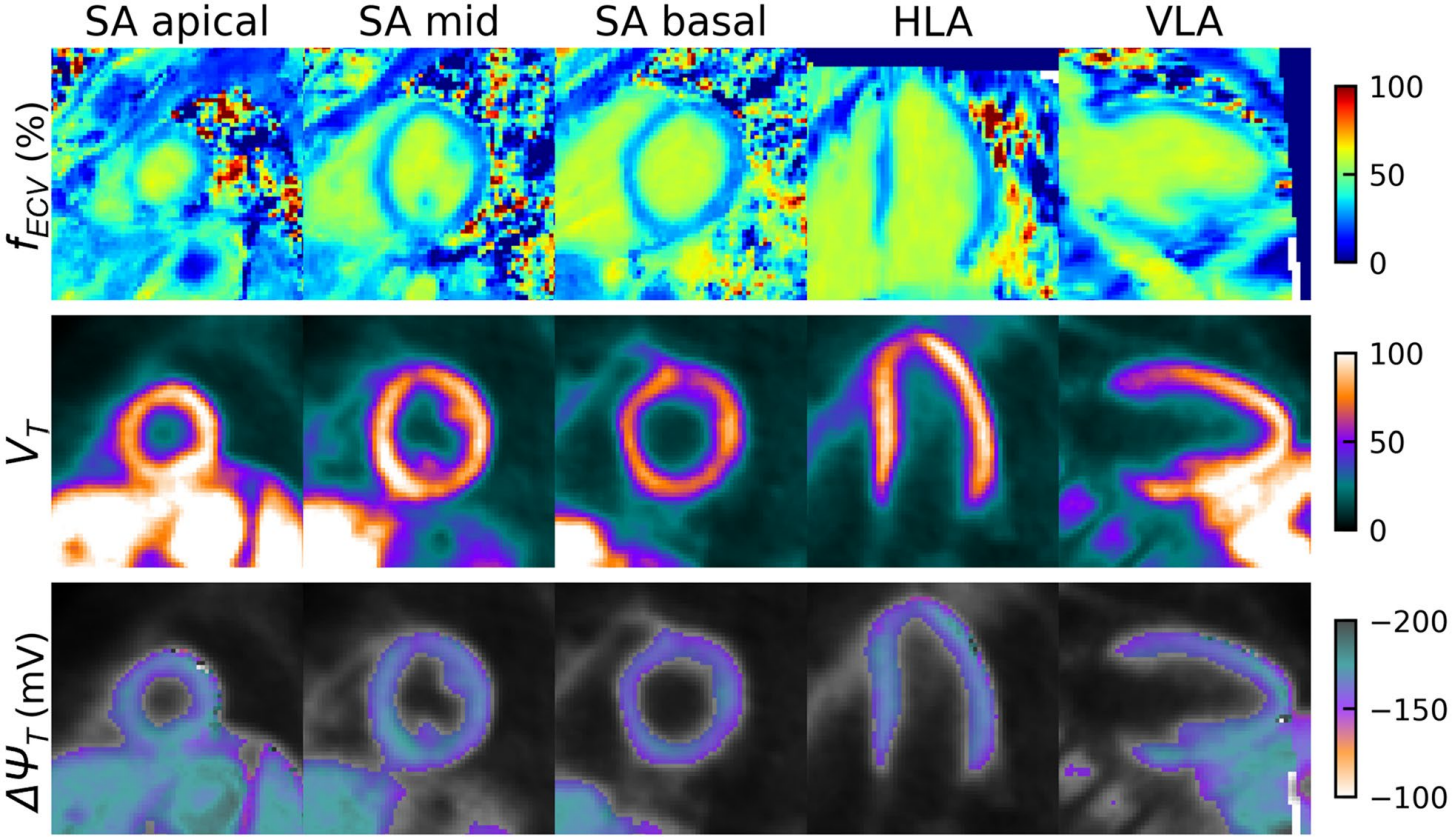
Extracellular volume fraction (*f*_*ECV*_), [^18^F]-FTPP^+^ volume of distribution (*V*_*T*_), and myocardial membrane potential (*ΔΨ*_*T*_) mapping results from a representative subject. Results are shown for representative slices in the apical, mid, and basal regions of the left ventricle in the short-axis view as well as horizontal and vertical long-axis views

Figure 4 shows the extracellular volume fraction, tracer volume of distribution, and membrane potential mapping results from the proposed method from the same representative subject. Overall, the values of the extracellular volume fraction, tracer volume of distribution, and membrane potential within the myocardium were visually in the range expected for a healthy subject across the different regions of the heart.

**Fig. 5:**
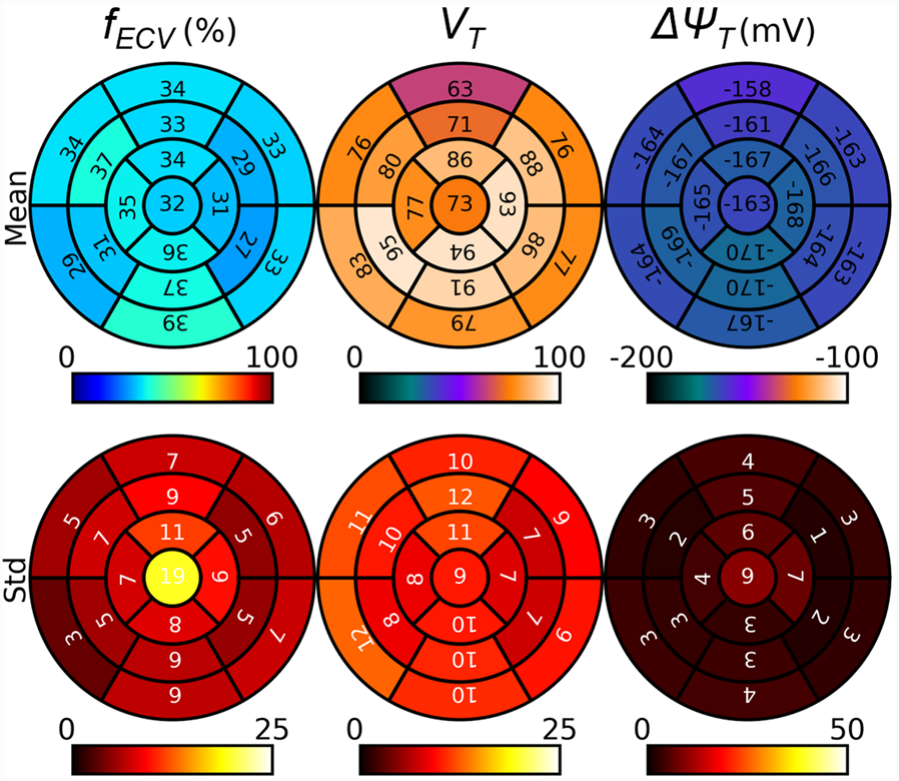
Bull’s eye plot of extracellular volume fraction (*f*_*ECV*_), [^18^F]-FTPP^+^ volume of distribution (*V*_*T*_), and myocardial membrane potential (*ΔΨ*_*T*_) from a representative subject. The mean and standard deviation are shown for each segment

Figure 5 and Supplementary Fig. 1 show the bull’s eye plot of extracellular volume fraction, tracer volume of distribution, and membrane potential. The extracellular volume fraction values were overall similar across the different regions of the heart and in the expected range for all three subjects. Some regions, particularly in the apical/basal and septal/inferior regions, showed higher than the expected mean (e.g., > 30.4% [26]) and standard deviation of extracellular volume fraction, which is presumed to be due to errors from the processes of image registration and/or partial volume effect. The estimated tracer volume of distribution and membrane potential values were also overall similar across the different regions of the heart and in the expected range, except for one subject (S1) which showed overall lower tracer volume of distribution and membrane potential in magnitude compared to those from the other two subjects. The estimated whole-heart membrane potential was − 144.7 ± 3.5 mV, − 160.7 ± 5.3 mV, and − 165.8 ± 3.1 mV, for each subject, respectively.

## Discussion

In this work, we have, for the first time, presented a method for in vivo 3D cardiac membrane potential mapping using [^18^F]-FTPP^+^ PET/MR imaging. Utilizing a recently developed MR-based 3D extracellular volume fraction mapping method [12], we improved the previously developed membrane potential mapping method [10] for fully 3D myocardial membrane potential mapping in a free-breathing acquisition scheme. The newly developed method improves patient comfort since it does not involve any breath-holding scans to be performed, which can be difficult to perform especially in heart disease patients often with dyspnea (e.g., shortness of breath). Also, the proposed method allows the estimation of membrane potential on a voxel-wise level with voxel-specific estimations of extracellular volume fraction and tracer volume of distribution. Assessment of membrane potential in the whole heart on a voxel-wise level could be a useful tool for evaluating a variety of cardiac diseases where mitochondrial dysfunction plays a role, such as in the case of heart failure or chemotherapy-induced cardiotoxicity.

Our protocol relies on a bolus-plus-infusion protocol, where a primed infusion of [^18^F]-FTPP^+^ is used to establish a secular equilibrium of tracer concentration both in the myocardium and in plasma. The tracer volume of distribution can then be quantified as the equilibrium ratio of tracer concentration in tissue and in plasma and subsequently be related to the membrane potential. While close to secular equilibrium had been reached in this study, a perfect equilibrium state is difficult to reach within a reasonable infusion time for the [^18^F]-FTPP^+^ tracer. The bolus-plus-infusion protocol necessitates a constant infusion over two hours, which can be challenging to achieve in some patient populations. An alternative approach, e.g., the use of dynamic imaging following a bolus-only injection of [^18^F]-FTPP^+^ with a dual-time-window, may be useful for reducing the overall imaging time compared to two hours of full-dynamic imaging while avoiding the need to administer a constant tracer infusion.

Accurate measurement of the myocardial extracellular volume fraction is critical for accurate quantification of myocardial membrane potential [8–10]. The myocardial extracellular volume fraction can be measured in humans in vivo using CT or MR imaging with contrast agent injection [12, 22, 23, 27, 28]. Both methods utilize the administration of a contrast agent, which allows quantitative measurement of extracellular volume fraction by observing the accumulation of contrast agent in the extracellular space via scans before and after the administration of the contrast agent. At the present time, the CT-based method is not widely used in practice due to the limited sensitivity of tissue contrast and radiation exposure. The MRI-based extracellular volume fraction mapping method is a non-invasive, non-irradiating, clinically accepted method [23, 26] that has been validated through histopathology [22]. The myocardial membrane potential mapping may be further improved by utilizing an improved myocardial extracellular volume fraction mapping method [29].

The current work has two limitations. First, since the study was designed to show the feasibility of the proposed method, the performance of the proposed method was validated through in vivo studies with a relatively small number of healthy subjects (*n* = 3). In our study cohort, we observed good inter-subject agreement between two of the three subjects, whereas one of the three subjects had a clearly less negative membrane potential. The subject did not have any known underlying cardiac disease or family history that may be related to the cause. Potentially, subjects with a depolarized membrane potential could be followed up to assess whether the observed abnormal membrane potential may be related to a later cardiac disorder. Studies with a larger cohort of healthy subjects and patients are necessary in the future to help assess the accuracy and reproducibility of the proposed method, to enable the establishment of a better-defined range of membrane potential for assessment, and to evaluate the value of the proposed method in clinical settings. Second, a simple combination of rigid and non-rigid image registration methods was utilized for motion correction and image registration in this work. A more sophisticated image registration method may be utilized for improved motion correction and alignment between PET and MR images for more accurate parameter estimation. Additional future work may include multi-site/multi-vendor validations of the proposed method and findings from this work.

## Conclusions

We have developed a non-invasive, free-breathing, in vivo 3D myocardial mitochondrial membrane potential mapping method using [^18^F]-FTPP^+^ PET/MRI. We have demonstrated the feasibility of the proposed method in three healthy volunteers.

## Supplementary Information

Supplementary material 1

## Data Availability

The datasets generated during and/or analyzed during the current study are available from the corresponding author on reasonable request.
