# Supplementary figures and images for: In vivo 3D myocardial membrane potential mapping in humans using PET/MRI
Source: EJNMMI Res. 2025 Jul 26;15:93. doi: 10.1186/s13550-025-01287-7 (PMC12297085; doi:10.1186/s13550-025-01287-7)

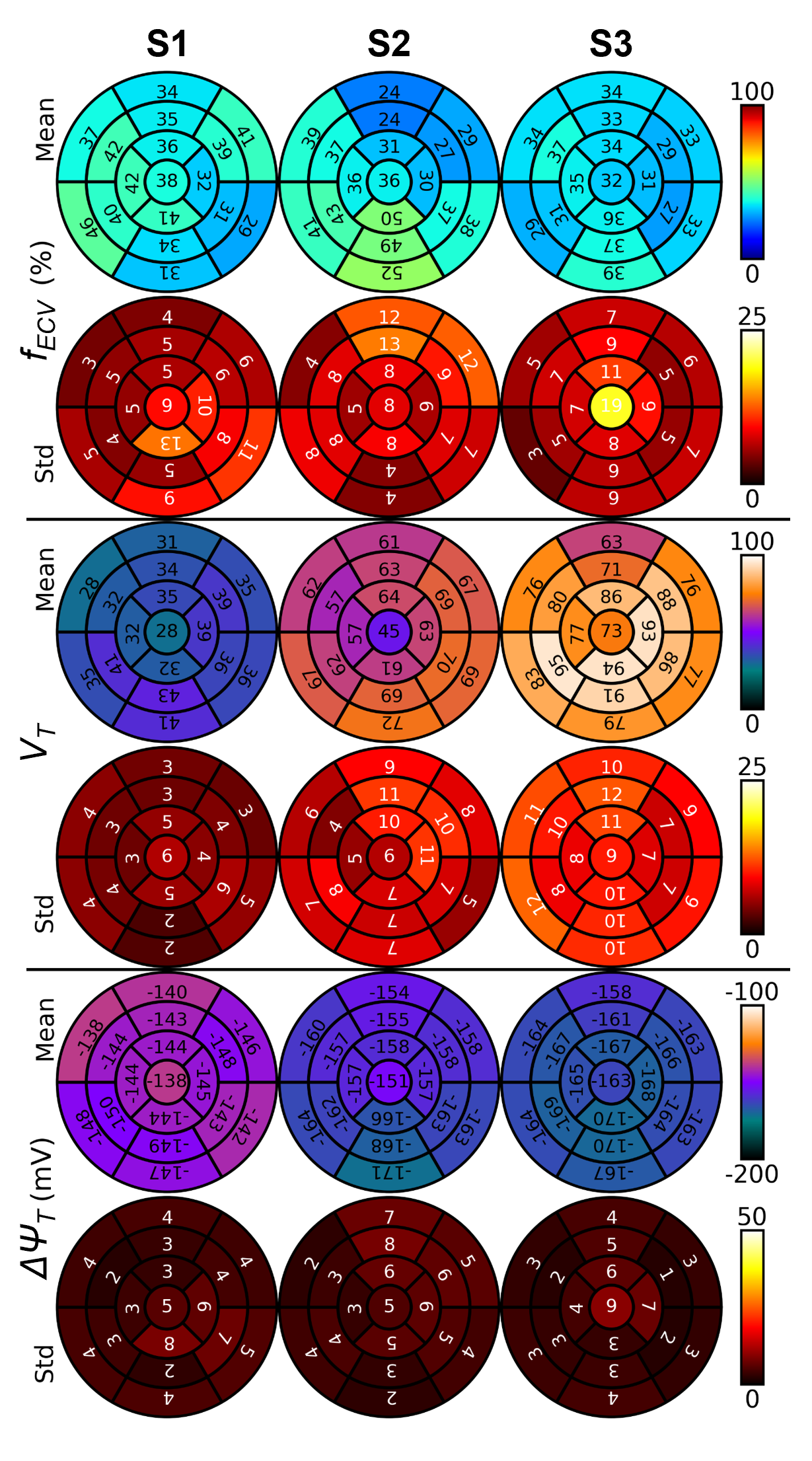

Supplement: Supplementary file 1 — Supplementary material 1 [file 13550_2025_1287_MOESM1_ESM.png]
